# Intergeneric hybrids inform reproductive isolating barriers in the Antarctic icefish radiation

**DOI:** 10.1038/s41598-019-42354-z

**Published:** 2019-04-12

**Authors:** Thomas Desvignes, Nathalie R. Le François, Laura C. Goetz, Sierra S. Smith, Kathleen A. Shusdock, Sandra K. Parker, John H. Postlethwait, H. William Detrich

**Affiliations:** 1 Institute of Neuroscience, University of Oregon, Eugene, OR 97403 USA; 2 Division des collections vivantes et de la recherche, Biodôme de Montréal/Espace pour la vie, 4777, Ave Pierre-De Coubertin, Montréal, QC H1V 1B3 Canada; 3 Department of Marine and Environmental Sciences, Marine Science Center, Northeastern University, Nahant, MA 01908 USA

## Abstract

Interspecific hybridization or barriers to hybridization may have contributed to the diversification of Antarctic icefishes (Channichthyidae), but data supporting these hypotheses is scarce. To understand the potential for hybridization and to investigate reproductive isolating mechanisms among icefish species, we performed *in vitro* fertilization experiments using eggs from a female blackfin icefish *Chaenocephalus aceratus* and sperm from a male of another genera, the ocellated icefish *Chionodraco rastrospinosus*. Sequencing of genomic and mitochondrial DNA confirmed the intergeneric hybrid nature of resulting embryos which successfully developed and hatched as active larvae at about four and a half months during the Antarctic winter. This result demonstrates the compatibility of gametes of these two species and the viability of resulting zygotes and larvae. Due to logistic constraints and the slow developmental rate of icefishes, we could not test for long-term hybrid viability, fertility, fitness, or hybrid breakdown. Analysis of our fishing records and available literature, however, suggests that the strongest barriers to hybridization among parapatric icefish species are likely to be behavioral and characterized by assortative mating and species-specific courtship and nesting behaviors. This conclusion suggests that, in long-lived fish species with late sexual maturity and high energetic investment in reproduction like icefishes, pre-mating barriers are energetically more efficient than post-mating barriers to prevent hybridization.

## Introduction

Icefishes (or Channichthyidae) have long been known by sailors and explorers of the Southern Ocean, but since the first scientific report of fish with “colorless blood” by Ditlef Rustad in 1927 and the validation in 1954 by Johan Ruud that icefishes live without hemoglobin in their blood^1^, these unique fish have fascinated evolutionary biologists. The 16 to 26 recognized species of icefish^2–6^ form an iconic lineage within the suborder Notothenioidei, which itself is a prime example of adaptive radiation^7,8^ and one of the rare examples of a vertebrate marine species flock^9–11^. The key evolutionary innovation of an anti-freeze glycoprotein (AFGP)^12,13^ and the unique environmental conditions of the Southern Ocean (e.g. vacant ecological niches and few predators)^2,14,15^ provide the foundation for understanding Notothenioid evolution, but how this group diversified to dominate the Antarctic ichthyofauna remains poorly understood. Due to the inherent difficulties of capturing and studying fish from isolated sub-Antarctic Islands and from the icy waters surrounding the Antarctic continent, only a few studies have examined Notothenioid diversity at the population genetic level^16–22^ or the potential for cryptic speciation among Notothenioids^23–25^.

Hybridization among diverging populations can boost speciation or prevent it, or alternatively, barriers to hybridization can promote species divergence^26–32^. The role of hybridization barriers to introgression in the Notothenioid adaptive radiation, however, is still unknown. Marino *et al*.^33^ detected signals of past genetic introgression and identified potential F1 hybrids among the three species of the icefish genus *Chionodraco*. To our knowledge, however, no other reports have identified individual fish as hybrids between two icefish species or, indeed, between any pair of Notothenioid species. Therefore, the potential for hybridization within Antarctic fishes remains uncertain.

To test the possibility of hybridization and to investigate the existence of hybridization barriers in Antarctic Notothenioids, we attempted *in vitro* fertilization between a female blackfin icefish *Chaenocephalus aceratus*, a species with a sequenced genome^34^, and a male ocellated icefish *Chionodraco rastrospinosus*. Although belonging to different genera that diverged from each other four to six million years ago^35^, these species, like all icefishes examined to date, have 24 chromosomes in a haploid set^36^, but neither hybridization nor genetic introgression have been recorded between them. Molecular genetic analyses confirmed that mature *C*. *aceratus* eggs were successfully fertilized by *C*. *rastrospinosus* sperm, and the resulting F1 hybrid embryos developed until at least two weeks post hatching, at which time the field season ended and the experiment had to be terminated. F1 hybrids were verified genetically by species-specific DNA polymorphisms. To our knowledge, this is the first successful attempt to make hybrids between different genera, or even different species, of Notothenioid fish. Results impact our understanding of barriers that may reproductively isolate icefish species and contribute to species divergence.

## Methods

### Collection and maintenance of icefishes

**Figure 1:**
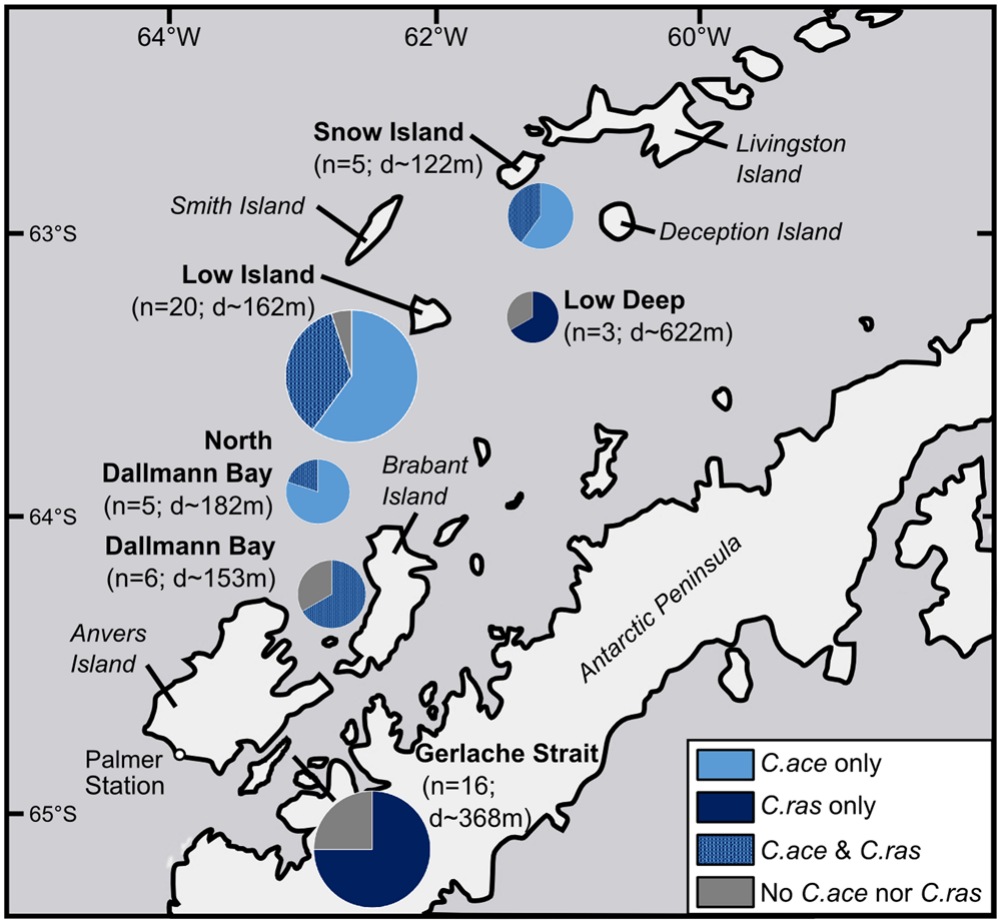
Capture records of *C*. *aceratus* and *C*. *rastrospinosus* specimens. Capture data are based on fishing operations performed aboard the *ARSV Laurence M Gould* in April-June 2016 along the WAP between Snow Island and Anvers Island. For each fishing location, the name of the fishing ground is in bold, and the number of tows performed at that given site during the season (n) and the average depth of these tows (d) are indicated below. The size of each pie chart is proportional to the number of tows performed at the site and pie sector size indicates the fraction of tows that captured *C*. *aceratus* only (light blue), *C*. *rastrospinosus* only (dark blue), both *C*. *aceratus* and *C*. *rastrospinosus* (intermediate blue), or neither *C*. *aceratus* nor *C*. *rastrospinosus* (grey).

Icefish specimens were collected, between April and June 2016, by bottom trawling from the *ARSV Laurence M*. *Gould* along the West Antarctic Peninsula (WAP), from Snow Island to Anvers Island (Fig. 1). At each fishing location, operators recorded, among other parameters, the GPS position of the trawl, the depth of the bottom, and a detailed list of the catch. Fish specimens were measured to the nearest millimeter and weighed to the nearest gram.

Fish were transferred immediately from the trawl net to the ship aquaria (1 m^3^ Xactics^TM^, Cornwall, Ontario, Canada) supplied with flow-through seawater with enhanced aeration as previously described^37^. After about a day or two at sea, live fish were transported to the aquatic facilities at Palmer Station, Antarctica, where they were maintained in 2.5 m^3^ flow-through seawater tanks at ambient temperatures of −1 to +1 °C following husbandry guidelines previously described^38^. Upon arrival on station, potentially sexually mature *C*. *aceratus* specimens of both sexes were injected intraperitoneally with Ovaprim® (0.5 ml.kg^−1^, Syndel, Ferndale, WA, USA), a gonadotropin-releasing hormone (GnRH) that stimulates gametogenesis in fish. The female used in this study received three injections over the course of one month. Specimens of *C*. *rastrospinosus* were not injected. All procedures were performed according to protocols approved by the Institutional Animal Care and Use Committees (IACUC) of the University of Oregon (#13-27RRAA) and of Northeastern University (#15-0207R).

### Collection of gametes and *in vitro* fertilization

Eggs were obtained from a gravid *C*. *aceratus* female by applying gentle pressure to its abdomen. Stripped eggs were collected in a clean stainless-steel bowl and kept chilled in ovarian fluid on ice to prevent activation until *in vitro* fertilization.

The testes of a mature *C*. *rastrospinosus* male were dissected out and rapidly cut into small fragments to let the semen exude into a petri dish.

For *in vitro* fertilization, given the low volume of sperm available, the whole testes and semen exudate of the *C*. *rastrospinosus* male were directly added to *C*. *aceratus* eggs. About 2.5 L of ambient, filtered, and UV-treated seawater was gradually added to dilute and activate the sperm. The mixture of eggs and sperm was gently agitated every 5 min for approximately 20 min. The negatively buoyant fertilized eggs were then rinsed three times in large amounts of filtered seawater to try to minimize egg adhesion.

### Care and sampling of embryos and larvae

The demersal embryos were transferred into two trays of a vertical incubator system (MariSource Inc., WA) with constant flow through of filtered, aerated, and UV-sterilized seawater at −1 to 0 °C. To prevent excessive microbial or fungal growth on egg chorions, embryos were surface disinfected by transfer to a solution of 150 ppm glutaraldehyde for 10 min. Disinfection began at three weeks post fertilization and was repeated every two weeks thereafter and until about one month prior to hatching.

Every week, a few embryos were randomly chosen for developmental and morphological observations and imaged using a dissection microscope. Because early embryos (i.e. from fertilization to 20–25 days post fertilization, dpf) were fragile and with opaque chorions, they were fixed and clarified in Stockard’s solution (50 ml formaldehyde, 40 ml glacial acetic acid, 60 ml glycerol and 850 ml distilled water) for 24–48 hours before observation. After 20–25 dpf, chorions were sufficiently transparent to permit direct microscopic observation of embryos. Beyond 50 dpf, embryos were dechorionated using fine forceps before observation. Every three to four weeks, a few embryos were sampled, their chorions punctured with a fine needle, and preserved in 70% ethanol for subsequent DNA analyses.

Most embryos hatched autonomously as larvae by 135 dpf. Embryos that were late to hatch were freed from their chorion by gently rubbing the egg with wet gauze. Hatchlings were maintained in large 2-L beakers of UV-treated filtered seawater placed in refrigerated incubators (−1 to 0 °C). Partial water changes (25–50%) were performed every two days. Zooplankton present in unfiltered seawater was periodically offered to the hatchlings to potentially initiate feeding behavior.

### Genetic analysis

To test whether the *C*. *aceratus* by *C*. *rastrospinosus* embryos were F1 hybrids or were gynogenetic haploids or diploids, we amplified and sequenced portions of three nuclear genes: *myh6* (*myosin*, *heavy chain 6*, *cardiac muscle*, *alpha*), *rho* (*rhodopsin*), and a portion of the intron of *rps7* (*ribosomal protein S7*) along with four mitochondrial genes: *mt-co1* (*cytochrome c oxidase I*, *mitochondrial*), *mt-cyb* (*cytochrome b*, *mitochondrial*), *mt-nd2* (*NADH dehydrogenase 2*, *mitochondrial*), and *mt-nd4* (*NADH dehydrogenase 4*, *mitochondrial*) from embryos at three different ages: 13 dpf (n = 3), 125 dpf (n = 3), and 132 dpf (n = 3). Primers used for gene amplification and sequencing are given in Supplemental Table 1. PCR reactions were performed as previously described^39^ and amplicon sequences were determined using ABI sequencing performed by GENEWIZ (Cambridge, MA, USA). Results were then compared to sequences deposited in NCBI and BOLD System^6^ to identify the species specific alleles. Representative sequencing results are provided in Supplemental File 1.

## Results

### *C*. *aceratus* and *C*. *rastrospinosus* have overlapping habitats

If two species are to hybridize, they must come in contact. Analysis of our 2016 fishing records for the capture of *C*. *aceratus* and *C*. *rastrospinosus* specimens revealed that, within our study area on the West Antarctic Peninsula (WAP) from Snow Island to Anvers Island (Fig. 1), *C*. *aceratus* and *C*. *rastrospinosus* were frequently collected together in the same trawl. *C*. *aceratus*, however, was exclusively captured at locations shallower than 200 m, whereas *C*. *rastrospinosus* was found within a wider bathymetric range with specimens caught as deep as 620–640 m. At locations shallower than 200 m where both species were captured (i.e. at Snow Island, Low Island, and North Dallmann Bay/Dallmann Bay), 40 ± 17% (mean ± standard deviation) of the tows simultaneously collected both *C*. *aceratus* and *C*. *rastrospinosus*. At locations deeper than 350 m (i.e. at Low Deep, and in the Gerlache Strait), only *C*. *rastrospinosus* specimens were captured. None of our trawl sites included the depth range 200–350 m, precluding a complete analysis of species distribution with respect to depth. Nonetheless, on the WAP, our capture data demonstrate that *C*. *aceratus* and *C*. *rastrospinosus* have partially overlapping spatial and bathymetric distributions and can be considered parapatric species.

### *C. aceratus* and *C. rastrospinosus* hybrids by *in vitro* fertilization

#### Parents and gametes

**Figure 2:**
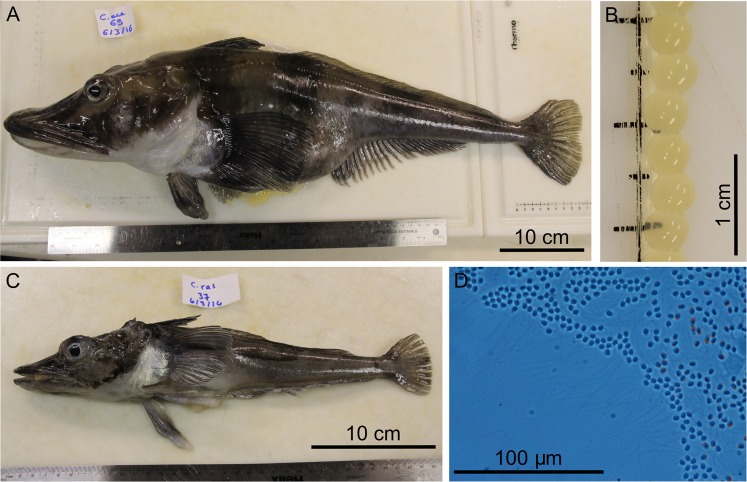
Parents and gametes. (**A**) The female *C*. *aceratus* that provided the eggs. (**B**) *C*. *aceratus* unfertilized eggs. (**C**) The male *C*. *rastrospinosus* that provided sperm. (**D**) Sperm of *C*. *rastrospinosus* observed under the microscope. Scale bars represent 10 cm in (**A**,**C**), 1 cm in (**B**), and 100 µm in (**D**).

On June 3^rd^, 2016, we collected approximately 20,000 ripe eggs from a single *C*. *aceratus* female (Fig. 2A,B). This female was caught in the vicinity of Low Island (Fig. 1) on May 9^th^, 2016, and with a total length (TL) of 68.0 cm and a weight of 2,721 g represented a large specimen for the species^3,40^. The female’s gonadosomatic index (GSI) was 27.85%. Before hydration and fertilization, her pale yellow eggs had a diameter of 3.89 ± 0.07 mm (mean ± SD) (Fig. 2B, Table 1) and mean wet and dry weights of 35.4 ± 0.9 mg and 8.1 ± 0.3 mg per egg, respectively (Table 1, n = 10).

**Table 1:** Characteristics of *C*. *aceratus* eggs.

State	Diameter (mm)	Wet Weight (mg)	Dry Weight (mg)
Unfertilized	3.89 ± 0.07	35.4 ± 0.9	8.1 ± 0.3
Fertilized	4.30 ± 0.12	48.5 ± 2.8	8.4 ± 0.4
Δ (%Δ)	0.41 (+10.6%)	13.1 (+37.1%)	0.4 (+4.7%)

Measurements were performed on 10 unfertilized eggs and 10 fertilized eggs. Delta is given as changes in fertilized eggs relative to unfertilized eggs.

The male *C*. *rastrospinosus*, which was caught at North Dallmann Bay (Fig. 1) on May 2^nd^, 2016, was much smaller than the female *C*. *aceratus* with a TL of 36.5 cm (Fig. 2C) and a weight of 386 g, values that are however above the averages for the species^3^. The GSI was estimated after semen release at 1 ± 0.5%. We examined a subsample of the *C*. *rastrospinosus* exuded semen at 600x magnification (Nikon Eclipse E800 microscope). Sperm heads were on average 3.04 ± 0.07 μm wide and 6.06 ± 0.07 μm long, and the flagellum measured 54.1 ± 0.1 μm for a total length of 60.1 ± 0.1 μm (n = 15; Fig. 2D).

Post fertilization, ten zygotes were measured and weighed. Results revealed a significant increase in diameter of ~10% (4.30 ± 0.12 mm) and ~37% in wet weight (48.5 ± 2.8 mg) (Table 1), consistent with hydration of fully mature eggs at fertilization and with previous reports of icefish egg sizes^41,42^.

#### Development of the embryos

**Figure 3:**
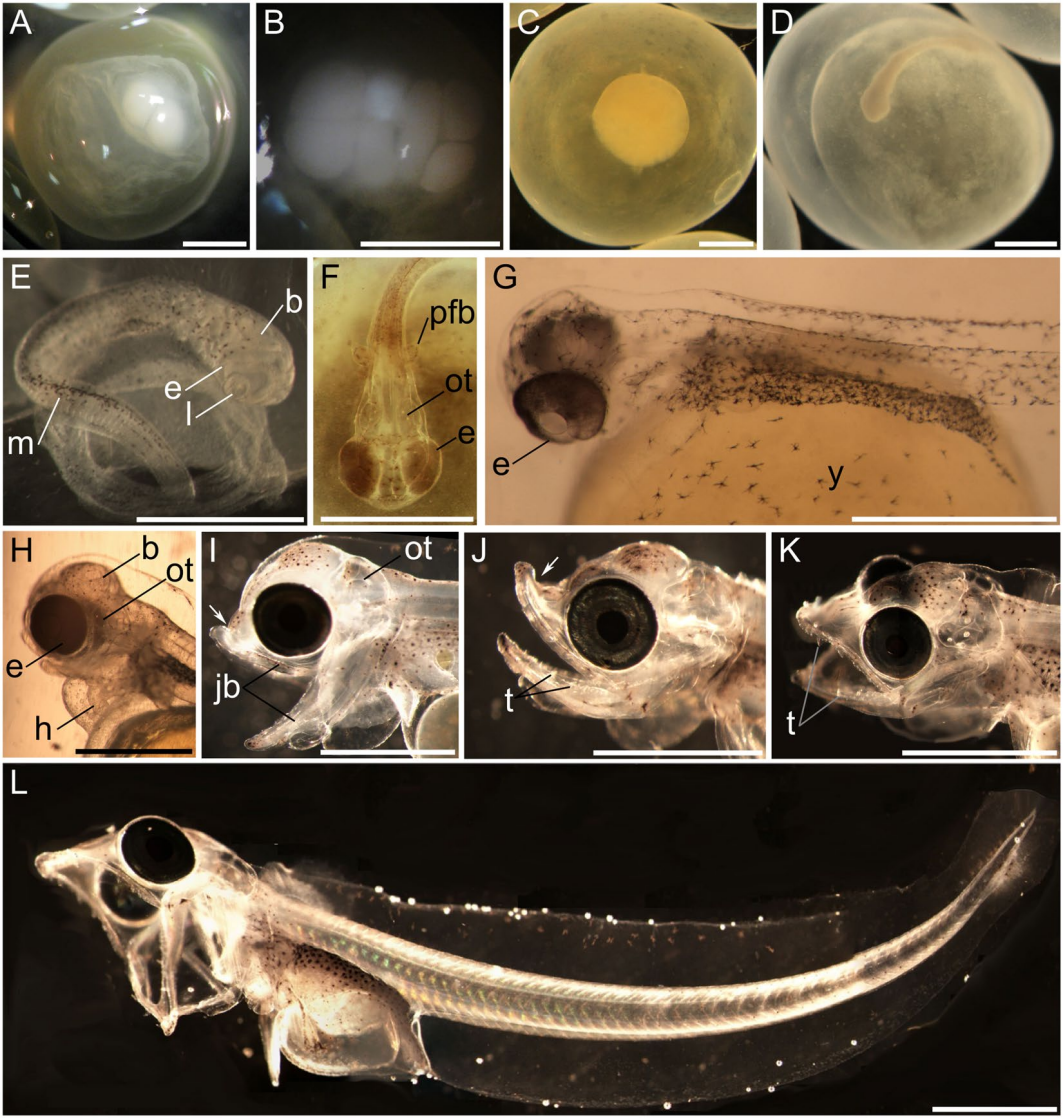
Development of the embryos from the *C*. *aceratus* by *C*. *rastrospinosus* intergeneric cross. (**A**) 1 dpf, 2-cell stage. (**B**) 2.5 dpf, 8-cell stage. (**C**) 15 dpf, blastula stage. (**D**) 38 dpf, early somite stage. (**E**) 48 dpf, brain regionalization stage. (**F**) 66 dpf, onset of retinal pigmentation. (**G**) 70 dpf, onset of vasculature formation. (**H**) 90 dpf, onset of cranio-facial skeletal formation. (**I**) 118 dpf, branchial arches evident. (**J**) 139 dpf, first fry. (**K**,**L**) 146 dpf, second fry stage. Scale bars represent 1 mm. Embryos in A–C were fixed with Stockard’s solution before imaging. Embryos in D–L were pictured live. Abbreviations: b, brain; e, eye; h, heart; jb, jaw bones; l, lens; ot, otoliths; pfb, pectoral fin bud; t, teeth; y, yolk sac. The white arrow in I and J points at the curvature of the developing snout.

Within one dpf, the first cell divided via meroblastic, discoidal cleavage to form two cells (Fig. 3A). Table 2 provides a detailed synopsis of the development of the embryos, and Fig. 3 shows images of key developmental stages. At 2.5 dpf, embryos possessed 8 cells (Fig. 3B) and by 4 dpf, at the early morula stage, about 128 cells had formed (Table 2). At the 128 cell stage, the sizes and shapes of blastomeres varied irregularly in each of our embryos. By 15 dpf, embryos were at the blastula stage (Fig. 3C). In contrast to *N*. *coriiceps* embryos^43^, the opacity of the chorion obscured visualization of the formation of the embryonic shield, but at 22 dpf, the embryonic axis became visible at the animal pole as the cloudiness of the chorion diminished (Table 2). Features of the embryonic head became recognizable around 38 dpf (Fig. 3D). By 48 dpf, delineation of the fore-, mid-, and hindbrain became apparent (Fig. 3E). Simultaneously, eyes with lenses (Fig. 3E), otoliths, and small rounded pectoral fin buds became visible (not visible in Fig. 3E). Melanophores began to decorate both the sides of the body, the dorsal side of the notochord, and the top of the yolk (Fig. 3E) (Table 2).

**Table 2:** Developmental stages of the embryos from the *C*. *aceratus* and by *C*. *rastrospinosus* intergeneric cross.

Age (dpf)	Stage	Developmental features
0	Fertilization	
1	2-cell stage	
4	Early morula stage	128-cell stage.
15	Blastula stage	Blastoderm of several thousand cells on top of the yolk.
22	Late gastrula stage	Embryonic shield clearly visible as a narrow streak.
38	Early somite stage	Otic vesicle visible.
48	Brain regionalization stage	Formation of the brain; eyes with lenses visible and otoliths in otic vesicles; initiation of cranio-facial features; first melanophores on the head, the sides of the body and the yolk; pectoral fin buds visible.
62	First movements	First embryonic twitching.
66	Onset of retinal pigmentation	Initiation of eye pigmentation.
70	Vasculature formation	Eyes ~80% pigmented; increase in melanophore number; vitelline vein visible.
78	Heart beating	First heart beats (3 beats.min^−1^); eyes fully pigmented.
90	Cranio-facial development	Cranio-facial skeletal elements identifiable.
98	Iridescent eyes	Eyes iridescent; snout begins to bend upwards.
110	Pigmentation stage	Increased melanophore numbers; snout growing upwards.
118	Branchial arches formation stage	Formation of the branchial arches; first teeth development.
125	Differentiation of the caudal fin	First actinotrichia in the caudal fin.
132	Hatching	Peak of hatching; first fully formed teeth.
139	First fry stage	Snout extending forward, becoming flatter.
146	Second fry stage	First rays in pectoral fins; increased number of teeth; snout becoming flat.

At 62 dpf, embryos first exhibited twitching movements of the tail (Table 2). Eyes began to pigment by 66 dpf (Fig. 3F) and were about 80% pigmented by 70 dpf (Fig. 3G). Between 66 and 70 dpf, melanophores increased in density on the sides of the body and on the yolk (Fig. 3F,G). The vitelline vein was also visible, extending from the bottom of the yolk to the heart (not visible on Fig. 3G). By 78 dpf, the heart had begun to beat at ~3 beats.min^−1^, and eyes were fully pigmented (Table 2). At 90 dpf, skeletal elements supporting the upper and lower jaws had formed, and the heart ventricle and atrium had enlarged (Fig. 3H). At 118 dpf, the fourth branchial arches had formed (not visible on Fig. 3I), and sharp teeth had begun to grow from the upper and lower jaws as the snout started to curve upwards (Fig. 3I,J). Actinotrichia began to appear in the caudal fin at 125 dpf (Table 2).

Two periods of hatching were observed, the first between 109 dpf and 123 dpf (~25% of embryos) and the second between 129 dpf and 136 dpf (~75% of embryos). The hatching peak occurred at 132 dpf (~4.5 months). Embryos from these two hatching periods did not present any noticeable morphological differences. All embryos were densely covered with melanophores, and their snouts continued to grow upward for two weeks (Fig. 3J). By the end of the experiment, at 146 dpf, snouts began to extend rostrally (Fig. 3K,L), the number of oral teeth increased (Fig. 3K), and actinotrichia had formed in the pectoral and caudal fins.

#### Embryos from the *C*. *aceratus* and *C*. *rastrospinosus* cross were true F1 hybrids

**Figure 4:**
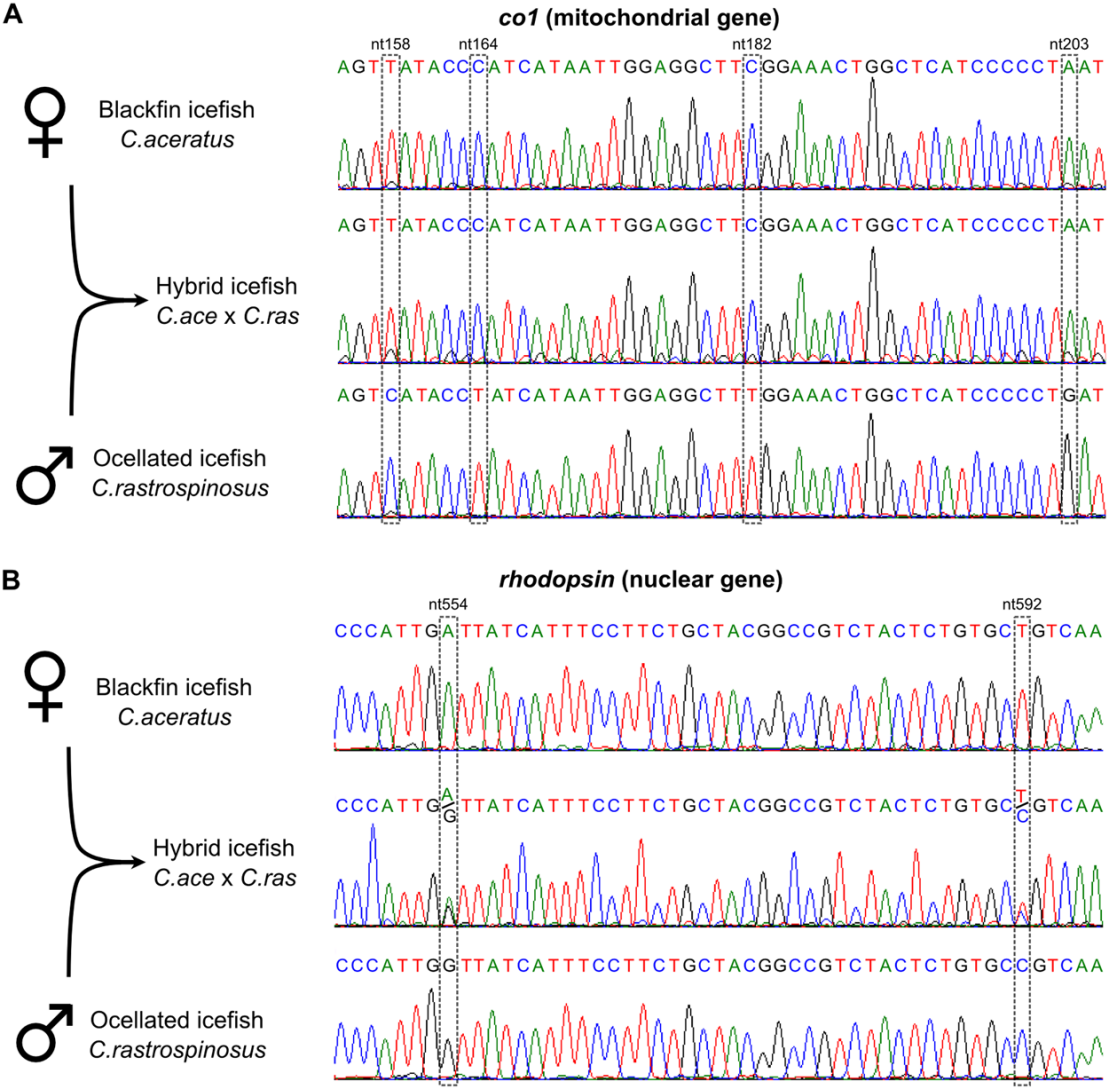
DNA sequencing confirms the hybrid status of intergeneric cross embryos. (**A**) A representative result of Sanger sequencing for the mitochondrial gene *mt-co1* identified only the maternal allele in experimental embryos. (**B**) In contrast, Sanger sequencing results for the nuclear gene *rhodopsin* demonstrated the presence of both the maternal and the paternal alleles in all individual embryos in approximately equal amounts, indicating heterozygosity. Positions of the SNPs in the sequences are given with respect to the maternal alleles.

To test whether embryos from the intergeneric cross were true F1 hybrids and not gynogenetic haploid or gynogenetic diploid embryos, we sequenced portions of four mitochondrial genes and three nuclear genes from both parents and from nine embryos of three different ages. Results showed that for the mitochondrial genes *mt-co1*, *mt-cytb*, *mt-nd2*, and *mt-nd4*, all nine embryos tested possessed only alleles with single nucleotide polymorphisms (SNPs) corresponding to those in the *C*. *aceratus* mother and none from the *C*. *rastrospinosus* paternal alleles. For example, for the mitochondrial *mt-co1* gene, maternal and paternal genotypes differed at 17 sites, and for all 17, embryos showed only the maternal *C*. *aceratus* allele (Fig. 4A, Supplemental Table 2). Similar results were found for the mitochondrial genes *mt-cytb*, *mt-nd2*, and *mt-nd4* (Supplemental Table 2). We conclude that the intergeneric cross embryos possessed only mitochondria from the *C*. *aceratus* mother, a result consistent with both models: hybrid origin and gynogenetic origin.

Results from genotyping nuclear genes, however, differed from results for mitochondrial genes. Using DNA from the same nine embryos tested for mitochondrial DNA, we amplified and sequenced portions of three nuclear genes, *myh6*, *rho*, and *rps7*. In the region examined, *myh6* had one SNP that differed between the *C*. *aceratus* mother and the *C*. *rastrospinosus* father, a T vs. C at position 85. Of the nine embryos examined, eight of nine had both the maternal T and the paternal C allele, one showed only the paternal C allele, and none had only the maternal T allele (Supplemental Table 2). Similarly, both maternal and paternal alleles were found for *rho* (Fig. 4B, Supplemental Table 2) and for *rps7* (Supplemental Table 2). Taken together, the embryos were heterozygous for all nuclear genes tested. Therefore, we conclude that the embryos were true F1 hybrids between the *C*. *aceratus* female and the *C*. *rastrospinosus* male.

## Discussion

*In vitro* fertilization of *C*. *aceratus* eggs by *C*. *rastrospinosus* sperm produced intergeneric F1 hybrid offspring that can develop at least until hatching. This unprecedented experimental outcome suggests a genetic and developmental potential for hybridization between icefish species and that hybridization might occur in nature. Because hybridization can in rare cases lead to new species^26–31^, our results suggest the possibility that hybridization contributed to the generation of novel icefish species. Limitations inherent to our experiment prevent us from evaluating this hypothesis. Our data, however, allow us to comment on the mechanisms that prevent these species, and probably other icefishes, from hybridizing in the wild. Based on the seminal work of Ernst Mayr^44^, hybridization barriers fall into two broad classes: pre-mating barriers that inhibit mating between species, and post-mating barriers that impact either the survival or the fertility of hybrids.

### Post-mating hybridization barriers

Post-mating hybridization barriers between species can occur through the inability of male gametes to fertilize oocytes, by lethality of the zygote or pre-reproductive juveniles, or by inhibition of the spread of hybrid genotypes in the population due to reduced hybrid fertility, sterility, or hybrid breakdown in the F2 generation. Our *in vitro* fertilization experiment rules out several types of post-mating barriers, including gametic incompatibility, because *C*. *rastrospinosus* sperm successfully fertilized *C*. *aceratus* eggs and produced embryos that developed past hatching. Indeed, our measurements of *C*. *rastrospinosus* spermatozoa head width (3.04 ± 0.07 μm) are compatible with their capacity to penetrate though the micropyle of *C*. *aceratus* oocytes, which is reported to measure ~5.6 μm in diameter^41^. Gametic compatibility and zygotic survival are also consistent with the recent divergence of the two species, within the last four to six million years^35^.

Our results also show that hybrid embryos develop at least up to hatching stage and produce viable larvae, therefore ruling out the reproductive barriers of zygotic mortality and early larval hybrid inviability. Furthermore, hybrid hatching peaked at 132 dpf (~4.5 months), which was well before hatching of *N*. *coriiceps* embryos (6 months post fertilization) cultured in similar conditions^43^ but almost identical to the 138 days reported for the hooknose icefish *Chionodraco hamatus*^45^, suggesting that the hybrid embryos developed at a normal rate. While these results demonstrate the absence of early and drastic genetic incompatibilities, we cannot rule out the possibility that hybrid larvae, juveniles, or adults would be significantly less fit than parental species in terms of survival, growth rate, or mating success^46^.

Unfortunately, logistic considerations prohibited the continued culture of hybrid larvae past five months, so it was not possible to raise hybrid animals until they reached developmental stages appropriate for testing F1 hybrid fertility or hybrid breakdown hypotheses. Antarctic icefishes require years of growth to reach sexual maturity, have low metabolic rates, yet make large energetic investments in reproduction^14,42,47^. Age at first spawning in *C*. *aceratus* has been predicted to be 13 to 14 years depending on location^40^, and age to maturity for *C*. *rastrospinosus* is uncertain but unlikely to occur before they reach five to eight years (32 to 37 cm TL)^42,48^. Should hybrid icefish offspring die before sexual maturity, display reduced fertility, be sterile, or show compromised fitness compared to both parental species, the gametic wastage by the parental species would be energetically and ecologically disadvantageous. Thus, establishment of pre-mating barriers among sympatric and parapatric species is a more efficient mechanism to prevent wasteful reproductive events from even occurring^49^.

### Pre-mating isolating barriers

Several different factors can contribute to pre-mating isolating barriers, including temporal isolation, ecological isolation, and behavioral isolation.

Temporal isolation occurs when two species reproduce at different times of the day or year. Our knowledge of the daily timing of icefish reproduction is limited to the *C*. *hamatus*, which in captivity appears to reproduce at night^45^. Whether *C*. *aceratus* and *C*. *rastrospinosus* reproduce at similar or different times of the day, is unknown. With respect to annual timing of reproduction, during our 2016 field season (mid-April to mid-June), we simultaneously identified in early June a male *C*. *rastrospinosus* with fluent sperm and gravid *C*. *aceratus* females that were in late vitellogenic stages. Our field data thus suggest that *C*. *rastrospinosus* and *C*. *aceratus* have overlapping reproductive seasons, in agreement with reported reproductive periods of March to May for *C*. *rastrospinosus* and from April to June for *C*. *aceratus* along the WAP^42,50^. While these two periods overlap, they may, however, differ sufficiently to inhibit cross reproduction. Therefore, temporal isolation may be a reproductive barrier reducing opportunities for hybridization between these two icefish species.

Ecological isolation occurs when two species inhabit different territories or do not reproduce in the same areas. Our capture records indicate that, in our WAP study area, *C*. *aceratus* and *C*. *rastrospinosus* are parapatric species and co-occur in at least some areas (Fig. 1). These observations are consistent with data in the literature that unambiguously indicate that *C*. *aceratus* and *C*. *rastrospinosus* have overlapping spatial and bathymetric distributions^2,51,52^. Near Bouvetøya, some *C*. *aceratus* nests have been observed at a depth of 140–150 m on flat and muddy to silty bottoms^53^. Mating grounds for *C*. *rastrospinosus* have not, to our knowledge, been documented. Therefore, while *C*. *aceratus* and *C*. *rastrospinosus* are parapatric species, different favored spawning grounds could constitute an ecological isolating barrier to hybridization. Such sites could differ in depth, slope, local bottom type, or associated benthic epifauna for example.

Behavioral isolation occurs when mating preferences and courtship differs between species. Icefishes are possibly behaviorally isolated through pre-reproductive courtship behaviors, including assortative mating and nesting.

Assortative mating and courtship may constitute a strong reproductive barrier. A female *C*. *aceratus* which is usually large may not find in a smaller and morphologically different male *C*. *rastrospinosus* the qualities or secondary sexual characters of a desired mate compared to male *C*. *aceratus*. Similarly, due to physical differences, a male *C*. *rastrospinosus* may not initiate courtship with a *C*. *aceratus* female, or the *C*. *aceratus* female may not reciprocate courtship by the *C*. *rastrospinosus* male. In the only directly observed instance of intraspecific reproduction in icefishes, it appeared that the male *C*. *hamatus* “infrequently moved close to the female to gently prod the [female’s] swollen abdomen using his snout. The female responded to such behavior with rapid movements of her caudal fin”^45^. Similar pre-reproductive behavior between sexes also occurs in the Antarctic naked dragonfish *G*. *acuticeps*^54^, but observations of intraspecific courtship for *C*. *aceratus* or for *C*. *rastrospinosus* are lacking. Therefore, we cannot predict how strong assortative mating is between these two icefish species or whether courtship behavior is incompatible and thus constitute a reproductive isolating barrier. In addition, if hybridization does occur in nature, both parental species might perceive sexually mature hybrids as less fit or less attractive than conspecifics, or they may display behavioral dysfunctions (e.g. unadapted courtship), and assortative mating may then evolve adaptively by reinforcement and strengthen existing behavioral barriers between species^55–58^.

Nesting behavior can also be an important factor influencing partner choice and mating success if nest types differ between species^59,60^. All icefishes are considered to reproduce only by external fertilization and some species^45,61–63^, including *C*. *aceratus*^53^ (Table 3), display nesting behavior, with males apparently preparing nests into which females deposit their eggs for fertilization, and DeWitt’s icefish, *Chionobathyscus dewitti*, brood their embryos on their pelvic fins^64^ (Table 3). The nests of *C*. *aceratus* are shallow depressions on muddy to silty bottoms^53^, whereas those of the spiny icefish *Chaenodraco wilsoni* and *Pagetopsis macropterus* are prepared on clean drop-stones^61–63^. Therefore, it seems unlikely that a female *C*.*aceratus* would be incited to deposit her eggs on a drop-stone prepared by a male *C*. *wilsoni* or *P*. *macropterus*, or brood eggs on her pelvic fins with a male *C*. *dewitti*. We do not know, however, if *C*. *rastrospinosus* males display significant nesting behavior, and, if they do make nests, whether or not they have the potential to attract female *C*. *aceratus* and induce egg laying. Therefore, given limited available data, we cannot predict the role of nesting in behavioral isolation between *C*. *aceratus* and *C*. *rastrospinosus*.

**Table 3:** Parental care features in icefishes.

Species	Reproductive behavior	Caring parent	Ref.
*Chaenocephalus aceratus*	Nest on mud/silt bottom with pebbles.	Male guarding	^53^
*Chaenodraco wilsoni*	Nest on high flat stone.	Male guarding	^61, 62^
*Chionobathyscus dewitti*	Egg clutches wrapped around pelvic fin.	Female carrying	^64^
*Chionodraco hamatus*	Nest on mud/silt bottom with pebbles.	Female guarding	^45^
*Pagetopsis macropterus*	Nest on clean stone.	Unknown	^63^

Taken together, our current knowledge on temporal, ecological, and behavioral characteristics of icefish reproduction, albeit incomplete, suggests the existence of multiple pre-mating isolation barriers that would limit successful mating between *C*. *aceratus* and *C*. *rastrospinosus* in nature. Among pre-mating barriers, behaviors associated with assortative mating, courtship, and nesting among icefishes are likely to act as the strongest reproductive isolating barriers. Whether these barriers are strong and restrictive or weak and permissive are subjects for future research.

### Hybridization in the icefish radiation

Marino *et al*.^33^ demonstrated past and probably continuing intrageneric hybridization between the three species of the *Chionodraco* genus. Using microsatellite genetic markers, they identified potential F1 hybrids between *C*. *rastrospinosus* and *C*. *hamatus* and between *C*. *rastrospinosus* and Myer’s icefish *C*. *myersi*. They also obtained evidence of mostly unidirectional genetic introgression from *C*. *rastrospinosus* into the other two *Chionodraco* species. Therefore, between these three closely related icefish species, hybridization barriers are still permissive and F1 hybrids may survive and can backcross with one of the parental species. Marino *et al*.^33^ proposed that past and present spatial distribution and likeliness of encounter between these species could have influenced the strength of hybridization barriers between them and could explain the observed imbalanced gene flow. Repeated contact between the sympatric and circum-Antarctic *C*. *hamatus* and *C*. *myersi* would result in strong hybridization barriers between them, whereas reduced contact between either species and the WAP-restricted *C*. *rastrospinosus* should reduce the need for strong hybridization barriers, thereby facilitating gene introgression from *C*. *rastrospinosus* into *C*. *hamatus* and *C*. *myersi*^33^. To our knowledge, no other signal of natural hybridization or genetic introgression in icefishes has been demonstrated. Potential hybridization among icefishes in nature, or among other notothenioid taxa, however, has not received much attention and may be frequent but underappreciated. Our experimental demonstration of a genetic and developmental potential for intergeneric hybridization between *C*. *aceratus* and *C*. *rastrospinosus*, together with the work of Marino *et al*.^33^, thus raise the possibility that interspecific hybridizations among icefishes occur in nature, and could generate novel species, provided that pre- and post-mating isolating barriers are non-restrictive.

## Conclusions

Signs of hybridization and genetic introgression were previously found between three congeneric *Chionodraco* species^33^, but no icefish hybrids between genera have yet been reported. Using field, experimental, and literature resources, we probed the potential for hybridization between icefish species and the barriers that would prevent inter-specific reproduction. The generation of *C*. *aceratus* and *C*. *rastrospinosus* hybrid larvae by *in vitro* fertilization demonstrates that the gametes of these two non-congeneric species are compatible, and that the resulting hybrid zygotes and embryos are viable at least until two weeks post hatching. Our short field seasons with respect to the extended time icefishes require to become reproductively mature currently precludes testing for long-term hybrid viability, hybrid sterility, potential hybrid breakdown, or the evaluation of fitness in hybrids compared to parental species. Analysis of fishing records from our studies and those of others, coupled with literature on the physiology and behavior of icefish reproduction leads to the conclusion that behaviors associated with assortative mating, courtship, and nesting among icefishes are likely to act as the strongest reproductive isolating barriers. Additional *in situ* and/or captive observations of spontaneous reproductive and brooding events are needed to help understand the importance of pre-mating reproductive barriers between icefish species. Broad scale population genetic analyses are also required to understand the extent of hybridization and genetic introgression among icefish species and to determine the role of hybridization in icefish species diversification.

## Supplementary information

Supplementary Files

Supplementary Data

## Data Availability

All data generated or analyzed during this study are included in this published article (and its Supplementary Information files).
