# Supplementary material for: Intergeneric hybrids inform reproductive isolating barriers in the Antarctic icefish radiation
Source: Sci Rep. 2019 Apr 12;9:5989. doi: 10.1038/s41598-019-42354-z (PMC6461676; doi:10.1038/s41598-019-42354-z)
Supplement: Supplementary file 1 — Supplementary Files [file 41598_2019_42354_MOESM1_ESM.docx]

# Intergeneric hybrids inform reproductive isolating barriers in the Antarctic icefishes radiation

Thomas Desvignes^1*#^, Nathalie R. Le François^2*#^, Laura C. Goetz^3^, Sierra S. Smith^3^, Kathleen A. Shusdock^3^, Sandra K. Parker^3^, John H. Postlethwait^1^, H. William Detrich III^3#^

1. Institute of Neuroscience, University of Oregon, Eugene OR 97403, USA
2. Division des collections vivantes et de la recherche, Biodôme de Montréal/Espace pour la vie, 4777, Ave Pierre-De Coubertin, Montréal, QC, H1V 1B3, CANADA
3. Department of Marine and Environmental Sciences, Marine Science Center, Northeastern University, Nahant, MA 01908 USA

* These authors contributed equally

^#^ Correspondence to [desvignes@uoneuro.uoregon.edu](mailto:desvignes@uoneuro.uoregon.edu); [nle_francois@ville.montreal.qc.ca](mailto:nle_francois@ville.montreal.qc.ca); [w.detrich@northeastern.edu](mailto:w.detrich@northeastern.edu);

# Additional Files

Additional Table 1: Primer sequences used for the genetic testing of hybrid status.

Additional Table 2: Detailed Sanger sequencing results for testing the hybrid status of intergeneric cross embryos.

Additional File 1: Representative Sanger sequencing traces for all seven studied genes in the *C. aceratus* mother, *C. rastrospinosus* father, and intergeneric cross embryos.
